# Editorial: Hierarchical Materials for Advanced Energy Storage

**DOI:** 10.3389/fchem.2020.601947

**Published:** 2020-11-12

**Authors:** Guanglin Xia, Tengfei Zhou, Xuebin Yu

**Affiliations:** ^1^Department of Materials Science, Fudan University, Shanghai, China; ^2^Institute for Superconducting & Electronic Materials, University of Wollongong, Wollongong, NSW, Australia

**Keywords:** hierarchical structure, hydrogen storage, electrochemical energy storage, interface, metal hydrides

It is urgent to search clean and renewable energy sources for maintaining the economic growth of modern society. In order to realize the versatile, clean, and efficient use of renewable energy, developing efficient energy storage materials and devices is of critical importance. To date, hydrogen storage and electrochemical energy storage are two main types of energy storage systems. Building hierarchical structures has been widely demonstrated to be an effective in advancing various energy storage materials owing to the unique physical and chemical properties induced by tuning their particle sizes, phases, and pores. Hierarchical structures offer several key advantages for advancing hydrogen storage materials and electrochemical energy storage materials, including: (i) the increased surface/contact area per unit mass, which could realize the fast diffusion and adsorption of active species; (ii) the tuned interface structures, which could promote the chemical reaction at the interface toward advanced energy storage performance; (iii) the accommodation of the mechanical strain, which could alleviate the structural damage upon the energy storage process and hence result in superior cycling stability. In this special issue, all the published papers could promote our understanding of the mechanism behind the improvement of energy storage materials via building hierarchical structures.

In this topic, Kim et al. reported the synthesis of binder-free cathode based on Fe foam modified with FeS_2_ for application in thermal batteries. The work of Zhang et al. demonstrated that, when applied in supercapacitors, three-dimensional core-branch α-Fe_2_O_3_@NiO/Carbon cloth exhibits high areal capacitance and stable cycling performance due to the reduction of contact resistance and the free-standing structure of the flexible electrode. The flexible and boron-Doped carbon nanotube film built by Wang et al. presents good rate capability and excellent cycling performance when used as a flexible anode in Li ion batteries. Xia et al. demonstrated the potential application of hierarchical structured electrocatalysts for overall water splitting. Moreover, Liu et al. presented a brief review about the progress of electrocatalytic hydrogen production induced by hierarchical porous molybdenum carbide-based nanomaterials and Guan et al. reviewed the progress of advanced supercapacitor materials using two-dimensional transition metal oxide and hydroxide-based hierarchical architectures.

In the field of hydrogen storage, Ali et al. revealed that the catalytic role of K_2_NbF_7_ on the dehydrogenation performance of LiAlH_4_ could be mainly attributed to in-formed NbF_4_, LiF, and K or K-containing phases during heating for hydrogen storage while Li et al. investigated the influence of various Al sources on the dehydrogenation behavior of LiBH_4_. According to the work of Chen et al., when adopting nanoporous Ni-based alloy as templates, the hydrogen storage performance of LiBH4 could be significantly improved owing to the reduction of particle size. In addition, Sun et al., reviewed the recent progress on improving the hydrogen storage performance of MgH_2_ using transition metals and carbon materials. Finally, as the Guest Editors of this topic issue, we would like to express our gratitude to all the authors for their valuable contributions and all the referees for their hard work and kind help. We hope that this special issue could boost the readers' research interest in building hierarchical structures toward advanced energy storage performance.

## Author Contributions

All authors listed have made a substantial, direct and intellectual contribution to the work, and approved it for publication.

## Conflict of Interest

The authors declare that the research was conducted in the absence of any commercial or financial relationships that could be construed as a potential conflict of interest.

